
JOURNAL INFORMATION
==============================
NLM Title Abbreviation: ScientificWorldJournal
Journal ID: ScientificWorldJournal
NLM Journal ID: 101131163
Journal ID: TSWJ

The Scientific World Journal

ISSN: 2356-6140
EISSN: 1537-744X
Publisher: Wiley

ARTICLE INFORMATION
==============================
PMCID: PMC6009425
PMID: 29973829
DOI: 10.1100/tsw.2002.54
Article ID: 890673
Article version: 1
Subjects: Short Report

Precursor Structure of Cephalosporin Acylase: Insights into Auto-Proteolytic Activation in a New N-Terminal Hydrolase Family

Kim Youngsoo 1 3 *
Kim Sanggu 1
Earnest Thomas N. 2
Hol Wim G.J. 3 4
School of Chemical Engineering, Yeungnam University, Dae-Dong, Kyungsan 712-749, ;, Republic of Korea
Berkeley Center for Structural Biology, Physical Biosciences Division, MS6-2100, Lawrence Berkeley National Laboratory, Berkeley, CA 94720;, USA
Department of Biochemistry, Biomolecular Structure Center, University of Washington, P. O. Box, 357742, Seattle, WA 98195-7742, USA
Howard Hughes Medical Institute, University of Washington, P. O. Box, 357742, Seattle, WA 98195-7742, USA
*Youngsoo Kim: ykim1@yu.ac.kr
Publication date: 2002
Electronic publication date: 2002 May 22
Volume: 2
First page: 115
Last page: 116
Copyright: Copyright © 2002 Youngsoo Kim et al.
Copyright year: 2002
License: This is an open access article distributed under the Creative Commons Attribution License, which permits unrestricted use, distribution, and reproduction in any medium, provided the original work is properly cited.
License URL: https://creativecommons.org/licenses/by/3.0/

==============================
Advances in Gene Technology: The Genome and Beyond –
Structural Biology for Medicine (Proceedings of the 2002 Miami
*Nature Biotechnology* Winter Symposium)
*TheScientificWorld* 2002, 2(S2), 115–116
ISSN 1532-2246; DOI 10.1100/tsw.2002.54

### PRECURSOR STRUCTURE OF CEPHALOSPORIN ACYLASE: INSIGHTS INTO AUTO-PROTEOLYTIC ACTIVATION IN A NEW N-TERMINAL HYDROLASE FAMILY

Youngsoo Kim[‡,§,*], Sanggu Kim[‡], Thomas N. Earnest[⊥], and Wim G.J. Hol[§,||]
[‡]School of Chemical Engineering, Yeungnam University, Dae-Dong, Kyungsan 712-749, Korea; [⊥]Berkeley Center for Structural Biology, Physical Biosciences Division, MS6-2100, Lawrence Berkeley National Laboratory, Berkeley, CA 94720; [§]Department of Biochemistry, Biomolecular Structure Center, and [||]Howard Hughes Medical Institute, University of Washington, P. O. Box, 357742, Seattle, WA 98195-7742
*ykim1@yu.ac.kr

**INTRODUCTION.** Autocatalytic proteolytic cleavage is a frequently observed post-translational modification in proteins. Cephalosporin acylase (CA) is a recently identified member of the N-terminal hydrolase family that is activated from an inactive precursor by autoproteolytic processing, generating a new N-terminal residue, which is either a Ser or a Thr[1]. The N-terminal Ser or Thr becomes a nucleophilic catalytic center for intramolecular and intermolecular amide cleavages. The gene structure of the open reading frame of CAs generally consist of a signal peptide followed by the α-subunit, a spacer sequence, and the β-subunit, which are all translated into a single polypeptide chain, the CA precursor. The precursor is post-translationally modified into an active heterodimeric enzyme with α- and β-subunits, first by intramolecular cleavage and second, by intermolecular cleavage.

**METHOD.** The structure of the S1βA precursor CA was determined to 2.5 Å resolution (Fig. 1). In the course of the structure refinement, the Fo-Fc difference Fourier map, with all the spacer residues omitted for phase calculation, showed clear positive density for spacer sequence prior to adding any information of these backbone residues in the unbiased map. The 11 residues of the spacer from 159s to 169s (EGDPPDLADQG) were easily built into the Fo-Fc difference map using O and were refined using CNS to an Rcryst of 20.3% and an Rfree of 23.7%.

**RESULTS AND DISCUSSION**. We solved the first CA precursor structure (Fig. 1) from a class I CA from *Pseudomonas diminuta* (CAD) at 2.5 Å resolution[2]. A conserved water molecule, stabilized by four hydrogen bonds in unusual pseudo-tetrahedral geometry, plays a key role to assist the OG atom of Ser1β to generate a strong nucleophile (Fig. 2a), which could provide insight into the mechanism of intramolecular cleavage. Thus, we would propose a plausible mechanism for the intramolecular cleavage of precursor CA. Initially, the hydroxyl of Ser1β is assisted by the conserved water (WAT1 in Fig. 2a), which is stabilized by four hydrogen bonds in pseudo-tetrahedral geometry and may accept a proton from the hydroxyl group. As a result, the hydroxyl of Ser1β is precisely positioned for nucleophilic attack[3]. Eventually, this nucleophilic attack will result in a free N-terminal Ser1β and the carboxylate of Gly169s (Fig. 2b).

### REFERENCES

1. Murzin, A.G. (1996) Curr. Opin. Struct. Biol. 6(3), 386–394.
2. Kim, Y., Yoon, K.H., Khang, Y., Turley, S., and Hol, W.J.G. (2000) Structure 8**,** 1059–1068.
3. Xu, Q., Buckley, D., Guan, C., and Guo, H.-C. (1999) Cell 98, 651–661.

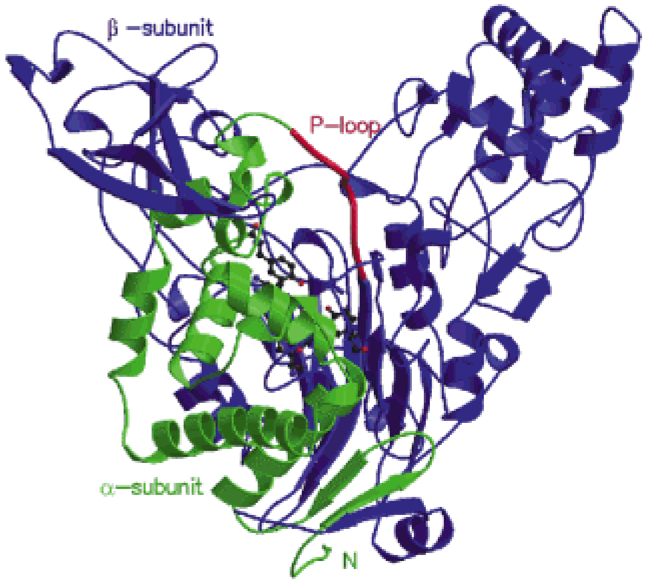

**FIGURE 1**. Structure of precursor CA. The precursor structure is a single chain protein, consisting of the α subunit of active CAD in green, an orange colored P-loop (spacer structure in loop form), and the β-subunit of CAD in purple. The side-chain pocket for binding the substrate glutaryl-7-aminocephalosporanic acid is represented by a ball-and stick model in the center[2].

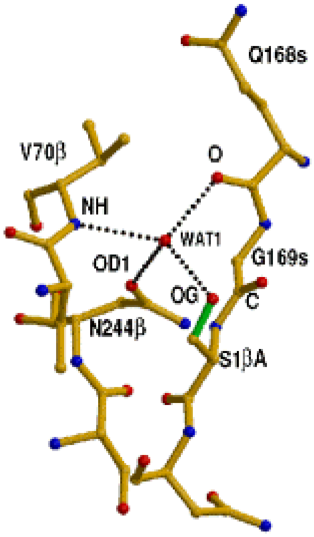

**a**

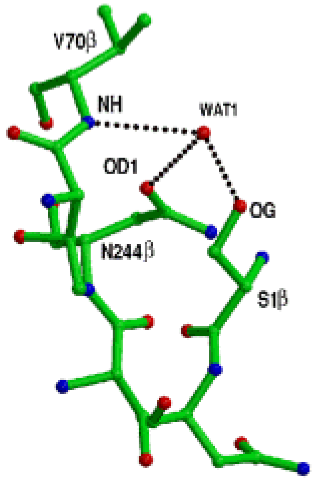

**b**

**FIGURE 2**. Hydrogen bonding of a conserved water in precursor CA and active CA. The dotted lines represents hydrogen bonds ranging in 2.8–2.9 Å. (a) The conserved water of precursor CA. The OG atom of Ser1β in the S1βA precursor CA was modeled after the torsional geometry of the OG atom of Ser1β residue of CA structure[2]. (b) The conserved water of CA. The conserved WAT1 is hydrogen bonded to 2 proton acceptors and 1 donor, which are also involved in chemical catalysis of CA[2].
